# The Efficacy of Intraosseous Access for Initial Resuscitation in Patients with Severe Trauma: A Retrospective Multicenter Study in South Korea

**DOI:** 10.3390/jcm13133702

**Published:** 2024-06-25

**Authors:** Youngmin Kim, Seung Hwan Lee, Sung Wook Chang, Yo Huh, Sunju Kim, Jeong Woo Choi, Hang Joo Cho, Gil Jae Lee

**Affiliations:** 1 Department of Trauma Surgery, Gachon University Gil Medical Center, Incheon 21556, Republic of Korea; skyofmay@gilhospital.com (Y.K.); surgeonrumi@gilhospital.com (S.H.L.); 2 Department of Traumatology, Gachon University College of Medicine, Incheon 21565, Republic of Korea; 3 Department of Thoracic and Cardiovascular Surgery, Dankook University Hospital, Cheonan 31116, Republic of Korea; changsw3@naver.com; 4 Division of Trauma Surgery, Department of Surgery, Ajou University School of Medicine, Suwon 16499, Republic of Korea; ermdhuhyo@gmail.com; 5 Department of Emergency Medicine, Yonsei University Wonju College of Medicine, Wonju 26426, Republic of Korea; mirage0124@naver.com; 6 Department of Emergency Medicine, Wonkwang University Hospital, Iksan 54538, Republic of Korea; dream-02@daum.net; 7 Department of Trauma Surgery, Uijeongbu St. Mary Hospital, College of Medicine, The Catholic University of Korea, Seoul 06591, Republic of Korea; surgeryman@catholic.ac.kr

**Keywords:** resuscitation, intraosseous, trauma centers

## Abstract

**Background/Objective:** In patients with severe trauma, intraosseous (IO) access is an alternative when intravenous (IV) access proves challenging. However, detailed insights into its utilization patterns and effectiveness are lacking. This study aims to evaluate the use and efficacy of IO access in hemodynamically unstable patients with trauma at level-1 trauma centers in South Korea. **Methods:** Data from six centers over 12 months were analyzed, focusing on patients with traumatic cardiac arrest or shock. Overall, 206 patients were included in the study: 94 in the IO group and 112 in the IV group. **Results:** The first-attempt success rate was higher in the IO group than in the IV group (90.4% vs. 75.5%). The procedure time in the IO group was also shorter than that in the IV group. The fluid infusion rate was lower in the IO group than in the IV group; however, the use of a pressure bag with IO access significantly increased the rate, making it comparable to the IV infusion rate. Further, regarding IO access, a humeral site provided a higher infusion rate than a tibial site. **Conclusions:** IO access offers a viable alternative to IV access for the initial resuscitation in patients with trauma, providing advantages in terms of procedure time and first-attempt success rate. The use of a pressure bag and a humeral site for IO access afforded infusion rates comparable to those associated with IV access.

## 1. Introduction

The rapid achievement of vascular access is crucial for life-saving interventions in patients with severe trauma. In cases of hemorrhagic shock, delays in the administration of fluids and blood products can have severe negative effects on patient outcomes. However, intravenous (IV) administration in emergency patients can be challenging. Shock can cause peripheral blood vessels to collapse, making IV access difficult. In these cases, intraosseous (IO) access can be a good alternative.

Since the first report of the use of therapeutic IO infusion in humans by Josefson in 1933, its application has been limited, primarily to pediatric resuscitation [1]. By the late 1990s, new mechanical IO devices began appearing on the market, leading to the increased adoption of IO access [2,3,4,5]. Recently, various guidelines, such as the Advanced Trauma Life Support (ATLS), Advanced Cardiac Life Support (ACLS), and European Resuscitation Council, recommend IO access as an alternative when traditional IV routes are difficult to establish [6,7,8]. IO access has shown good efficiency, especially in prehospital and combat environments where time and conditions do not favor IV insertion, and it provides an effective conduit for the administration of various agents, including radiological contrast agents and anesthetics [9,10,11].

Despite the increasing acceptance and utilization of IO approaches in emergency and trauma care, the literature comparing the outcomes of IO and IV approaches in patients with severe trauma, especially those experiencing traumatic cardiac arrest or hemodynamic instability, remains ambiguous. Although several studies have underscored the successful application of IO access, they report conflicting outcomes, and the volume of research is insufficient [12,13]. The use of IO injections has rapidly increased worldwide; however, it likely remains underused [14]. In South Korea, the trauma system has developed rapidly over the past decade, and the preventable mortality rate among patients with trauma has significantly improved [15]. However, there is limited research on the frequency and circumstances of IO injection use, as well as the manner of IO access adoption across different trauma centers. This study aims to investigate the implementation status of IO access in hemodynamically unstable patients with trauma admitted to level-1 trauma centers in South Korea and analyze the effectiveness of IO access compared with the traditional IV access route.

## 2. Materials and Methods

### 2.1. Study Design and Patient Selection

This retrospective analysis was conducted over 12 months, from 1 October 2020 to 30 September 2021, across six level-1 trauma centers in South Korea. Patients transferred to level-1 trauma centers during this period were included, with a specific focus on those who experienced traumatic cardiac arrest or traumatic shock upon arrival. Traumatic shock was defined as a condition where the patient had a systolic blood pressure below 90 mmHg or signs of inadequate perfusion, such as altered mental status, oliguria, or peripheral cyanosis. Patients with evident signs of death upon arrival, such as rigor mortis, and contraindications for IO access, including fractures, infections, compartment syndrome at the intended site of the procedure, previous IO access attempts within 24 h, severe deformity or damage at the procedure site, and pregnancy, and ages ˂18 years were excluded. All vascular access procedures, including IV and IO, were performed upon hospital arrival and not in the prehospital setting.

### 2.2. Classification

**Figure 1:**
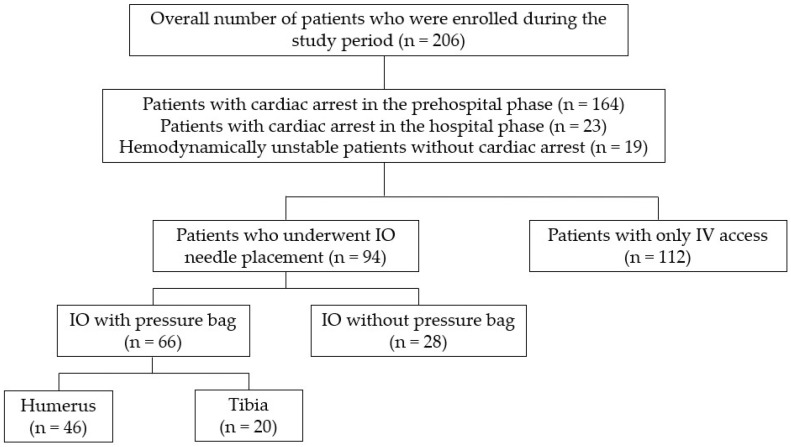
The Flowchart of Patient Distribution.

Patients were classified into the IO or IV groups based on the initial access route used upon arrival at the emergency department. The IO group included patients who primarily received IO access first due to the difficulty in obtaining IV access. In some cases, both IO and IV access was established simultaneously by multiple practitioners attempting line access. Both routes were utilized for resuscitation in these instances, but patients were categorized into the IO group. Only the variables associated with the IO access route were evaluated for scientific analysis. For subgroup analysis, the IO group was divided into two groups based on whether a pressure bag was used: pressure bag and under-gravity groups. The pressure bag group was further divided into humeral and tibial groups based on the anatomical site of IO access (Figure 1).

### 2.3. Outcome Measures and Techniques

The outcome measures focused on various aspects, including the time to apply each technique, the total volume and rate of fluid infusion, the administration of medications and blood products, the site of application, the number of attempts, and the problems encountered. The EZ-IO^®^ System (Teleflex, Morrisville, NC, USA) was used for IO injection in either the humerus or tibia, compared with traditional peripheral or central venous access methods.

### 2.4. Statistical Analysis

Statistical analysis was conducted using SPSS software version 20.0, where continuous variables were compared using Student’s *t*-test, and categorical variables were compared using the chi-square or Fisher’s exact test. Statistical significance was set at a *p*-value of ≤0.05.

## 3. Results

In this study, 206 patients were analyzed, including 94 and 112 in whom IO and IV access was established, respectively. In the IO group, 24 received IO access only, and 70 received both IO and IV access.

### 3.1. Demographics and Clinical Characteristics

The basic demographics for the entire patient cohort are detailed in Table 1.

The average patient age was 51.7 ± 21.6 years, with the IV group being older than the IO group (54.6 ± 20.4 years vs. 48.2 ± 22.7 years, respectively). The injury severity score was higher in the IV group than in the IO group, indicating more severe injuries in the IV group. However, among the 187 patients who presented with pre- or in-hospital cardiac arrest and 19 who were hemodynamically unstable, those from the IO group had a higher proportion of pre- or in-hospital cardiac arrests.

### 3.2. Access Times and Success Rates

The time from emergency department arrival to line access was longer in the IO group (5.9 ± 5.3 min) than in the IV group (2.9 ± 2.3 min, *p* < 0.001). However, the procedure’s duration was shorter in the IO group (1.0 ± 0.1 min) than in the IV group (1.9 ± 1.4 min, *p* < 0.001), with the IO group also having a higher first-attempt success rate (90.4% vs. 75.5%). Intergroup differences in the rates of crystalloid infusion were not significant; however, blood and vasopressors were administered at higher rates in the IV group. The occurrence of problems during their use did not differ significantly between the two groups (Table 2).

### 3.3. Infusion Rates and Pressure Bag Use

Regarding infusion rates, the rate was higher in the IV group (53.0 ± 38.5 mL/min) than in the IO group (39.0 ± 38.6 mL/min, *p* = 0.012). However, the use of a pressure bag for fluid infusion via IO access significantly increased the fluid infusion rate (45.9 ± 42.9 mL/min), compared with the “under gravity” condition in the IO group (20.8 ± 11.0 mL/min, *p* = 0.005), as shown in Table 3.

Additionally, on comparing the pressure bag subgroup of the IO group with the IV group, no significant difference in fluid infusion rates was observed (45.9 ± 42.9 mL/min vs. 53.0 ± 38.5 mL/min, *p* = 0.256).

### 3.4. Subgroup Analysis of Infusion Rates by Anatomical Location

When the pressure bag subgroup was divided into the humeral and tibial groups according to the anatomical location of IO access and the infusion rates were compared, the fluid infusion rate at the humerus site was found to be significantly higher than that at the tibia site (53.7 ± 47.9 vs. 27.8 ± 19.3, *p* < 0.023) (Table 4).

## 4. Discussion

This study examined the efficacy of IO access compared with IV access for initial resuscitation in severely injured patients transported to six level-1 regional trauma centers in South Korea. Our findings indicate that IO access was associated with a shorter procedure time and higher first-attempt success rate than IV access. Additionally, the infusion rate through the humerus when using a pressure bag was comparable to that when using IV access.

Rapid line access is crucial in the initial resuscitation phase of patients with trauma with cardiac arrest or hemorrhagic shock. Achieving rapid line access for initial resuscitation can be facilitated through a combination of factors, including a short procedure duration, high first-attempt success rates, and high infusion rates. Compared with IV access, IO access is known for its shorter procedure duration and higher first-attempt success rate [13,16,17,18,19,20]. This advantage stems from the non-collapsible nature of the IO cavity and the simplicity of the technique, which requires less learning time [21,22,23,24,25,26]. Recent meta-analyses of prehospital patients with trauma have shown significantly higher first-attempt success rates and reduced mean procedure times for IO access than for IV access [12]. Moreover, the mean procedure time was significantly shorter in the IO group than in the IV group. A review of three studies reported that the success rate of EZ-IO^®^ ranged between 87% and 96% [13]. In a study comparing IO access with a central venous catheter, IO access was found to have a high success rate (85%) and a low mean procedure time (2.0 min) [24]. The results of this study, supporting previous research, demonstrate a procedure time that is twice as short as that of IV and a success rate exceeding 90%.

In this study, IO access was associated with significantly lower infusion rates than IV access. In an injured porcine model study, Warren et al. reported that peripheral IV access was linked to a higher infusion rate than IO access [27]. However, a literature review of various studies comparing the infusion rates associated with IV and IO access revealed highly variable results [13]. This is because the infusion rate is influenced by various factors. The rate of fluid administration is governed by physical principles. According to Poiseuille’s law, the radius and length of the tube through which the fluid flows markedly affect the flow rate [28,29]. Thus, the size and length of infusion needles must be considered. Additionally, the viscosity of the fluid affects the flow rate, which means that the speed may vary depending on the type of fluid (such as blood or crystalloids) and the medication administered [30]. The increased resistance in the space where the fluid is injected can reduce the pressure difference and reduce the flow rate, leading to differences in speed depending on the anatomical location. This means the flow rate can change depending on the degree of peripheral vasoconstriction or the resistance of the medullary cavity of the long bones [31].

To assess the variation in the fluid infusion rate due to pressure differences, the IO infusion rates were compared with and without the application of a pressure bag. Using a pressure bag significantly increased administration speed, which was not inferior to that of IV access. The infusion flow rates associated with pressure bag use in IO administration are known to range from 30 to 70 mL/min, and our study reflects these results [32,33,34]. Increasing the pressure during blood transfusion can increase the possibility of hemolysis, indicating a need for further research into the safety of blood administration [34,35,36,37].

There may be a difference in pressure due to resistance at the anatomical location. This study demonstrated that the infusion rate was two times higher for administration through a humeral site than through a tibial site. Similar results were reported in cadaver studies. Pasley et al. measured the speed of IO infusion at different anatomical locations in 16 cadavers at a pressure of 300 mmHg [28]. They reported that the humerus received an average volume 1.8 times greater per unit time than the tibia, a rate consistent with our findings (humerus, 57.1 ± 43.5 mL/min; tibia, 30.7 ± 18.7 mL/min). Lairet et al. infused normal saline into 11 pigs, with the infusion at each site being for 10 min using a pressure bag, and determined that the flow rate through a proximal humeral site was higher than that through a proximal tibial site [38]. In contrast, Ong et al. compared the tibial and/or humeral IO flow rates for normal saline with and without pressure bag use in 24 patients [39]. They did not find a statistically significant difference in infusion rates between the proximal tibial site (165 mL/min) and the proximal humeral site (153 mL/min). A large-scale prospective study under controlled conditions is necessary.

This study had some limitations. Because this was a retrospective study, there were differences in characteristics between the two groups. Age, sex, and underlying diseases can affect bone density, thus posing potential limitations pertaining to our study population and possibly influencing infusion rates [28]. Additionally, considering the variables included in the previously mentioned physical laws, a more precise comparison could have been made if the length and radius of the device used, as well as the pressure, had been consistently controlled. Furthermore, this study lacks a description of the size, location, and number of IV (peripheral or central) lines to which IO access is compared. Finally, it has been reported that infusion via one anatomical site versus simultaneous infusion via two sites can produce a difference in speed [32]. After excluding patients in the IO group who were also administered IV, a comparison between the IO-only and IV groups should have been performed; however, this was not feasible because of the small number of patients.

Despite these limitations, this study is significant as it is the first to evaluate the multicenter usage status of IO access in South Korea. It demonstrates the practical feasibility of IO access for initial resuscitation in patients with trauma. Particularly, IO access can be considered a primary option for rapid access during trauma resuscitation. This is crucial in the prehospital setting and critical in-hospital scenarios, such as during cardiopulmonary resuscitation in patients experiencing or about to undergo cardiac arrest. In South Korea, the use of IO access by emergency medical technicians (EMTs) in the prehospital setting is legally limited, and it has not been widely adopted by physicians in hospitals. This study provides foundational data for the broader application of IO access in South Korea. It evaluates the use of IO access in realistic and practical environments, and future research should aim to validate and expand these findings in more controlled settings. This will further clarify the efficacy of IO access and encourage its adoption and utilization by EMTs and healthcare providers in South Korea.

## 5. Conclusions

During the initial fluid resuscitation of patients with trauma, IO access allows for fluid administration as quickly as IV access. IO access is a good alternative for hemodynamically unstable patients with trauma. However, large-scale prospective studies are required to further explore its efficacy and application.

## Figures and Tables

**Table 1:** The baseline characteristics of the study population.

	Total (*n* = 206)	IO Group (*n* = 94)	IV Group (*n* = 112)	*p*-Value
Age	51.7 ± 21.6	48.2 ± 22.7	54.6 ± 20.4	0.037
Sex				0.306
M	135 (65.5)	58 (61.7)	77 (68.8)	
F	71 (34.5)	36 (38.3)	35 (31.3)	
Mechanism of injury				0.012
Falls	96 (46.6)	50 (53.2)	46 (41.1)	
Pedestrian struck by a vehicle	37 (18.0)	20 (21.3)	17 (15.2)	
Motor vehicle crash	24 (11.7)	4 (4.3)	20 (17.9)	
Motorcycle or bicycle collision	22 (10.7)	8 (8.5)	14 (12.5)	
Blunt force assault	10 (4.9)	7 (7.4)	3 (2.7)	
Penetrating injury	6 (2.9)	1 (1.1)	5 (4.5)	
Others	11 (5.3)	4 (4.3)	7 (6.3)	
Transport mode				<0.001
Ground ambulance	184 (89.3)	90 (95.7)	94 (83.9)	
Helicopter	22 (10.7)	4 (4.3)	18 (16.1)	
Type of transfer				<0.001
Direct (primary transfer)	178 (86.4)	92 (97.9)	86 (76.8)	
Transfer (secondary transfer, interhospital)	28 (13.6)	2 (2.1)	26 (23.2)	
AIS				
Head and neck	3.2 ± 1.8	2.5 ± 1.4	3.8 ± 1.9	<0.001
Face	1.6 ± 0.7	1.6 ± 0.8	1.6 ± 0.7	0.913
Thorax	3.1 ± 1.5	2.7 ± 1.3	3.5 ± 1.5	<0.001
Abdomen/pelvis	3.0 ± 1.9	2.0 ± 1.4	3.3 ± 2.0	0.007
Extremity	2.9 ± 1.3	2.7 ± 1.3	3.1 ± 1.3	0.060
External	1.1 ± 0.5	1.1 ± 0.3	1.2 ± 0.7	0.609
ISS	21.5 ± 13.9	18.6 ± 11.3	23.9 ± 15.4	0.006
Initial mental status				0.217
Alert	5 (2.4)	1 (1.1)	4 (3.6)	
Verbal response	3 (1.5)	0 (0)	3 (2.7)	
Painful response	6 (2.9)	2 (2.1)	4 (3.6)	
Unresponsive	192 (93.2)	91 (96.8)	101 (90.2)	
Hemodynamic status				<0.001
Cardiac arrest in the prehospital phase	164 (79.6)	87 (92.6)	77 (68.8)	
Cardiac arrest in the hospital phase	23 (11.2)	3 (3.2)	20 (17.9)	
Hemodynamic instability without cardiac arrest	19 (9.2)	4 (4.3)	15 (13.4)	
Prehospital CPR	133 (64.6)	82 (87.2)	51 (45.5)	<0.001
Death in the TER	170 (82.5)	84 (89.4)	86 (76.8)	0.026
In-hospital mortality	202 (98.1)	93 (98.9)	109 (97.3)	0.627

Abbreviations: IO, intraosseous; IV, intravenous; ISS, injury severity score; CPR, cardiopulmonary resuscitation; TER, trauma emergency room.

**Table 2:** Comparison between the IO and IV groups.

	IO Group (*n* = 94)	IV Group (*n* = 112)	*p*-Value
Time from TER arrival to IO needle or IV catheter insertion (min)	5.9 ± 5.3	2.9 ± 2.3	<0.001
Procedure duration (min)	1.0 ± 0.1	1.9 ± 1.4	<0.001
Number of total attempts, *n* (%)			0.003
1	85 (90.4)	71/94 (75.5)	
2	9 (9.6)	18/94 (19.1)	
3	0	4/94 (4.3)	
4	0	1/94 (1.1)	
Administration, *n* (%)			
Crystalloid	76/78 (97.4)	95/100 (95.0)	0.469
Blood	9/76 (11.8)	52/100 (52.0)	<0.001
Vasopressor	25/71 (35.2)	53/100 (53.0)	0.029
Problems in use, *n* (%)			0.436
None	87 (92.6)	96/100 (96.0)	
Difficulty in injecting fluid or drugs	6 (6.4)	4/100 (4.0)	
Displacement after insertion	1 (1.0)	0 (0)	
Inappropriate insertion site, *n* (%)	6/76 (7.9)	NA	NA
Pressure bag use, *n* (%)	66 (70.2)	NA	
Fluid infused (mL)	446.1 ± 279.5	935.4 ± 687.5	<0.001
Infusion time (min)	16.8 ± 13.4	20.3 ± 11.3	0.044
Infusion rate (mL/min)	39.0 ± 38.6	53.0 ± 38.5	0.012
Transfusion (unit)			
RBCs	0.1 ± 0.4	2.0 ± 2.9	<0.001
FFP	0	0.8 ± 2.3	0.002
PLTs	0	0	NA

Abbreviations: IO, intraosseous; IV, intravenous; TER, trauma emergency room; RBCs, red blood cells; PLT, platelets; FFP, fresh frozen plasma; NA, Not Applicable.

**Table 3:** Comparison of fluid infusion rates with and without pressure bag use in the IO group between the IO and IV groups.

	IO Group			IV Group (*n* = 112)	*p*-Value ^1^
	Pressure Bag (*n* = 66)	Under Gravity (*n* = 28)	*p*-Value		
Fluid infused (mL)	485.0 ± 286.6	354.3 ± 242.7	0.028	935.4 ± 687.5	<0.001
Infusion time (min)	15.8 ± 13.0	18.9 ± 14.2	0.326	20.3 ± 11.3	0.016
Fluid infusion rate (mL/min)	45.9 ± 42.9	20.8 ± 11.0	0.005	53.0 ± 38.5	0.256

^1^ Compared to the “with pressure bag” scenario and the IV group.

**Table 4:** Comparison of fluid infusion rates according to insertion site in the pressure bag subgroup of the IO group.

	Humerus (*n* = 46)	Tibia (*n* = 20)	*p*-Value
Fluid infused (mL)	526.1 ± 281.0	390.5 ± 283.8	0.082
Infusion time (min)	15.5 ± 12.8	16.7 ± 13.9	0.734
Fluid infusion rate (mL/min)	53.7 ± 47.9	27.8 ± 19.3	0.023

## Data Availability

Dataset available on request from the authors.
